# Trainable segmentation for transmission electron microscope images of inorganic nanoparticles

**DOI:** 10.1111/jmi.13110

**Published:** 2022-05-11

**Authors:** Cameron G. Bell, Kevin P. Treder, Judy S. Kim, Manfred E. Schuster, Angus I. Kirkland, Thomas J. A. Slater

**Affiliations:** ^1^ Electron Physical Sciences Imaging Centre Diamond Light Source Oxfordshire UK; ^2^ Department of Materials University of Oxford Oxford UK; ^3^ Rosalind Franklin Institute Harwell Science and Innovation Campus Didcot UK; ^4^ Johnson Matthey Technology Centre Reading UK; ^5^ School of Chemistry Cardiff University Cardiff UK

**Keywords:** machine learning, scanning transmission electron microscopy, image segmentation, metal nanoparticles, transmission electron microscopy

## Abstract

We present a trainable segmentation method implemented within the python package ParticleSpy. The method takes user labelled pixels, which are used to train a classifier and segment images of inorganic nanoparticles from transmission electron microscope images. This implementation is based on the trainable Waikato Environment for Knowledge Analysis (WEKA) segmentation, but is written in python, allowing a large degree of flexibility and meaning it can be easily expanded using other python packages. We find that trainable segmentation offers better accuracy than global or local thresholding methods and requires as few as 100 user‐labelled pixels to produce an accurate segmentation. Trainable segmentation presents a balance of accuracy and training time between global/local thresholding and neural networks, when used on transmission electron microscope images of nanoparticles. We also quantitatively investigate the effectiveness of the components of trainable segmentation, its filter kernels and classifiers, in order to demonstrate the use cases for the different filter kernels in ParticleSpy and the most accurate classifiers for different data types. A set of filter kernels is identified that are effective in distinguishing particles from background but that retain dissimilar features. In terms of classifiers, we find that different classifiers perform optimally for different image contrast; specifically, a random forest classifier performs best for high‐contrast ADF images, but that QDA and Gaussian Naïve Bayes classifiers perform better for low‐contrast TEM images.

## Lay description

Measurement of the size, shape and composition of nanoparticles is routinely performed using transmission electron microscopy and related techniques. Typically, distinguishing particles from the background in an image is performed using the intensity of each pixel, creating two sets of pixels to separate particles from background. However, this separation of intensity can be difficult if the contrast in an image is low, or if the intensity of the background varies significantly. In this study, an approach that takes into account additional image features (such as boundaries and texture) was investigated to study electron microscope images of metallic nanoparticles. In this ‘trainable segmentation’ approach, the user labels examples of particle and background pixels in order to train a machine learning algorithm to distinguish between particles and background. The performance of different machine learning algorithms was investigated, in addition to the effect of using different features to aid the segmentation. Overall, a trainable segmentation approach was found to perform better than use of an intensity threshold to distinguish between particles and background in electron microscope images.

## 1 INTRODUCTION

Electron microscopy is frequently used to characterise inorganic nanoparticles and can be used to determine important characteristics such as size, shape^1^ and lattice strain.^2^ These properties can be related to other parameters such as catalytic activity,^3^ and additional processing can reveal 3D structure,^4^, ^5^ or can group similar nanoparticles according to their characteristics to facilitate 3D reconstructions (for each group).^6^ However, to correctly analyse nanoparticle images, the nanoparticles must be isolated, or segmented, from the background of the image. This alone is a challenge, as manually segmenting nanoparticles by hand is very time consuming, and often many nanoparticles must be segmented to produce statistically significant results.

Common methods of image segmentation include global and local thresholding, which rely exclusively on the intensity of an image's pixels. Global thresholding algorithms set a minimum or maximum intensity value across a whole image and pixel intensities above or below this are binarised to create a binary segmentation mask. Examples of these global algorithms include the Otsu,^7^ Yen^8^ and Li^9^ algorithms. Local thresholding, using algorithms such as Niblack^10^ and Sauvola,^11^ works similarly, but sets a threshold on a per pixel basis from the range of intensities in its local neighbourhood. The use of global and local thresholding methods is pervasive in the analysis of nanoparticle images,^12^, ^13^, ^14^ but any thresholding method is limited in the types of data that can be successfully segmented. When segmenting high‐contrast images, such as those obtained from high‐angle annular dark field (HAADF) images in the scanning transmission electron microscope (STEM), thresholding methods are often sufficient for accurate segmentation. However, segmentation of low‐contrast transmission electron microscope (TEM) images can be particularly challenging using intensity thresholding, as has been repeatedly shown in cryo‐electron microscopy.^15^ Local thresholding can be of particular use when intensity of illumination or thickness of any support material changes over an image, but may lose accuracy in extreme cases.

In many cases, the use of information beyond simple pixel intensities assists in segmentation, whether that is the detection of object edges,^16^ texture^17^ or specific shapes^18^ in images. This additional information can be extracted from images by convolving the image with a defined kernel (i.e. a matrix of values for pixelated images). Convolutional kernels assign a value to a pixel, given by the convolution of the local region around that pixel to a specific kernel structure (examples of which are described in Section 2), rather than using the pixel intensity alone. Segmentation can be performed from individual images that are the result of a convolution with a single kernel, or the pixel values of the output from multiple convolution kernels can be used. Identifying which kernels to use and which values correspond to which label (e.g. background vs. particle) can be done using a machine‐learning approach, whether that is using a neural network or the type of trainable segmentation presented here.

There has been significant interest in the use of convolutional neural networks to exploit additional image information for image segmentation.^19^, ^20^, ^21^ Neural networks are a set of machine‐learning algorithms based on a layered structure of artificial neurons, where each neuron operates on data received from a previous layer, before passing it to the next layer.^19^ The training process alters the weights that relate the neurons to one another, which improves the accuracy of the network. A convolutional neural network contains convolutional layers that consist of a set of learnable kernels.^22^ Training a convolutional neural network alters the convolutional kernels in each convolutional layer, in addition to the weights of each neuron, optimising the found features to segment the training data. Once trained, neural networks are very accurate on the trained data set, as their structure is specifically tailored to it. However, adapting a neural network to a different data set typically requires fine‐tuning the neural network and retraining. Neural networks also require large numbers of pre‐segmented images, to act as a ground truth, to train the neural network to become sufficiently accurate. The training process also takes a significant amount of time, making neural networks unsuitable for real‐time segmentation during image acquisition, unless the network has been pre‐trained on a similar data set.

A related approach to convolutional neural networks is known as trainable segmentation, which has been popularised by the WEKA segmentation available in ImageJ.^23^ Trainable segmentation uses pre‐defined convolutional kernels, rather than learning effective kernels when training a convolutional neural network. Initially, a number of pixels are manually labelled (e.g. background and particle). The labelled pixels and corresponding feature values are used to train a classifier, which defines boundaries between labelled sets of particle and background pixels, based on the intensities of the generated feature set. This produces a fully segmented image and a trained classifier,^24^ which can subsequently be used to classify further images. Trainable segmentation is typically more accurate than thresholding methods, as we will demonstrate in our later results, but less accurate than neural networks.^25^ However, compared to neural networks, the samples of training data needed for accurate classification is considerably smaller in trainable segmentation. The time to train the classifier is also shorter than the time needed to train a neural network, by several orders of magnitude.

There are existing implementations of trainable segmentation such as those in the ImageJ^23^ and Ilastik^26^ software packages, but these are typically standalone programs. We have implemented a trainable segmentation methodology in ParticleSpy, a python package for segmentation and analysis of HyperSpy^27^ signal objects. As ParticleSpy is written in python, it can be easily expanded upon and integrated into workflows, particularly those using the HyperSpy ecosystem.^27^

Previous studies have successfully used trainable segmentation to segment inorganic nanoparticles from electron microscopy data,^28^, ^29^ but these studies have not investigated the parameters used in trainable segmentation. To produce accurate and effective segmentation results, the mechanisms behind trainable segmentation, and the filter kernels and classifiers used, need to be understood. Here we investigate the effectiveness and similarity of the implemented filter kernels and the use cases and requirements for different classifiers. The accuracy of trainable segmentation is evaluated for different TEM image sets and compared to standard thresholding techniques.

## 2 METHODS

ParticleSpy is written in python, supported by commonly used packages including NumPy, Sci‐Kit Image^30^ and Sci‐Kit Learn.^31^ All classifiers in Sci‐Kit Learn can be used in ParticleSpy's trainable segmentation. Its user interface, however, only presents a selection of the most common classifiers.

**FIGURE 1:**
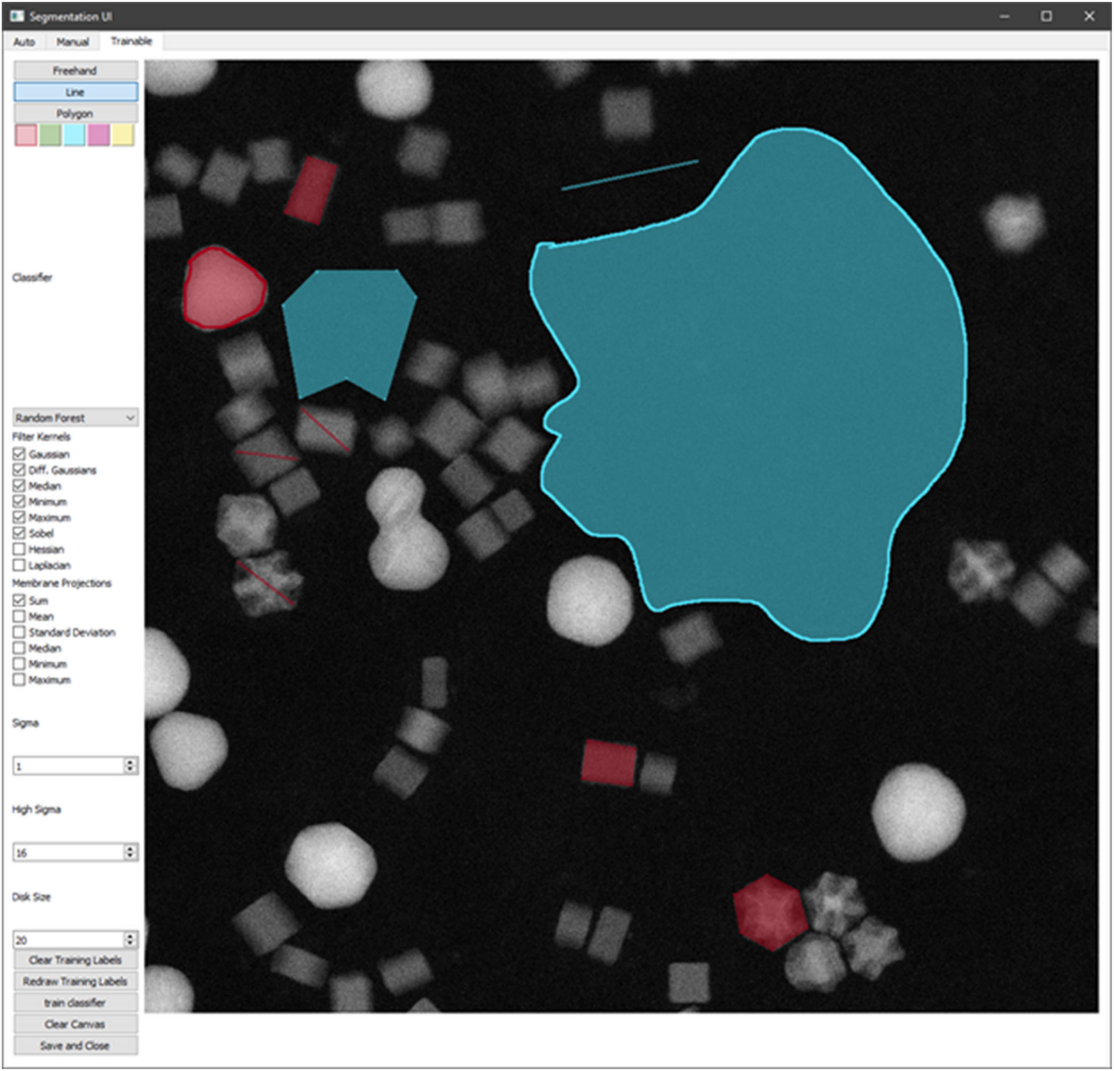
The ParticleSpy trainable segmentation user interface showing the use of line, polygon and freehand labelling tools. The classifier and filter kernels used for training can be specified using the menu on the left.

The User interface in ParticleSpy (Figure 1) can be used to perform basic thresholding, manual segmentation, or trainable segmentation and the algorithms and parameters for thresholding can be adjusted individually to adjust the segmentation.

The trainable segmentation interface enables labelling using freehand, line and polygon tools, and flood fills, for up to five different labels. The user interface allows images to be trained using the drawn labels, including retraining with updated labels if required. The filter kernels used can also be selected individually and have their parameters adjusted. Manually segmented images from external programs can also be used to produce labelled images for training. This was done for all four data sets examined using either GIMP^32^ or ImageJ.^23^ The primary user customisation of ParticleSpy is through its filter kernels and classifiers, which are detailed in the next two sections.

### 2.1 Filter kernels

**FIGURE 2:**
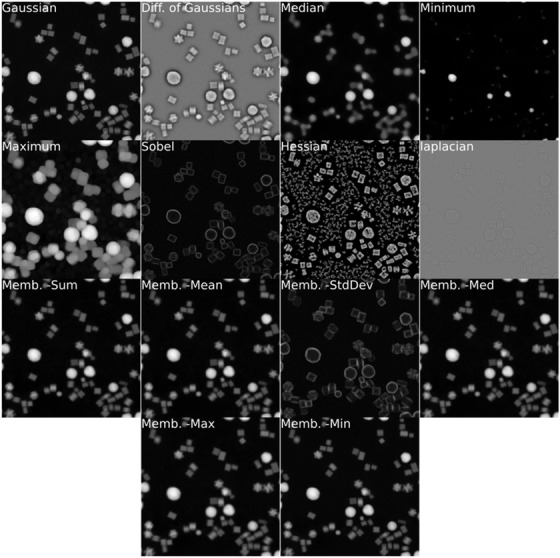
Output of labelled filter kernels used in ParticleSpy when applied to one of the PdPtNiAu ADF images.

Descriptions of each of the filter kernels found in ParticleSpy are given below. An example of the application of each filter kernel is shown in Figure 2.

The Gaussian filter kernel convolves the image with a matrix weighted to an approximate Gaussian. The difference of Gaussians filter kernel does this twice for two different sizes of Gaussians and calculates the difference between these.

The median, minimum and maximum filter kernels take the median, minimum and maximum values respectively within a given radius as the value of each pixel. The radius of each filter kernel can be provided as a user input or will default to 20 pixels in size.

The Sobel filter applies two first‐order edge detection filter kernels that highlight edges, or regions with a large gradient.^16^ The image is convolved with two matrices, highlighting horizontal and vertical gradients, which are combined to form a single image of edges.

The Laplacian filter kernel is a second‐order filter kernel that also detects edges in images, but is potentially less sensitive to noise than the Sobel filter. The Laplacian filter is a discrete approximation to the following equation:

(1) $$\nabla^{2}f\left(x,y\right)=\frac{\delta^{2}f}{\delta x^{2}}+\frac{\delta^{2}f}{\delta y^{2}},$$

where *f* is the function describing the image.

The Hessian filter kernel is another second‐order filter kernel that is used to find ridges, to form an approximated Hessian matrix. Elements of the Hessian matrix **H** are defined as

(2) $${\left(H_{f}\right)}_{i,j}=\frac{\delta^{2}f}{\delta x_{i}\delta y_{j}},$$

where *i* and *j* are the indices of the matrix.

Membrane projections are a group of filter kernels based on those in ImageJ. These kernels filter the image directionally to highlight membrane–like structures. A 19 × 19 identity matrix is convolved with the image at 30 different angles between 0° and 180°, then the 30 images produced are combined using six different methods, taking the sum, mean, standard deviation, median, maximum and minimum of the 30 values of each pixel. These produce six features, which are used for classification.

### 2.2 Classifiers

Classifiers are used in trainable segmentation to define boundaries between labelled sets of particle and background pixels to classify unlabelled pixels. The choice of a particular classifier can have a significant impact on the accuracy and segmentation time for a given data set. The four classifiers we have chosen to use in this study, based on their ease of use and higher accuracies, are outlined below.

#### 2.2.1 Random forest

Random forest classifiers^33^ function by fitting a number of decision trees, models which predict values from decision rules based on the training data, to subsamples of the training data. These subsamples are then averaged to improve accuracy and reduce overfitting, which improves the overall accuracy of the classifier.

#### 2.2.2 Nearest neighbours

The nearest neighbours classifier^34^ operates simply by storing instances of the training data, which the classification process uses to vote on each point requiring classification, where a simple majority vote decides the label of each pixel. This is calculated using the *K* nearest neighbours for each point, where *K* is a user‐defined integer.

#### 2.2.3 Gaussian Naïve Bayes

The Gaussian Naïve Bayes classifier^35^ operates using Bayes’ theorem, which relates the probability of a pixel label to its feature set. The probability is calculated for the training data by generating a model that tries to fit the training data of each label. The shape of this model is naively assumed to take the form of a Gaussian. It has very few adjustable parameters beyond changing the assumed distribution.

#### 2.2.4 Quadratic discriminant analysis

The quadratic discriminant analysis (QDA) classifier^36^ operates using a quadratic boundary to separate particle and background pixels, the distributions of which are predicted using Bayes’ rule. Due to the use of Bayes’ rule for initial prediction, if the distribution of two labels are distinct (i.e. no labels are present on the wrong side of the quadratic boundary), then the classifier produced is identical to that of the Gaussian Naïve Bayes. Similarly to that classifier, QDA does not require any parameter adjustment.

### 2.3 Filter kernel accuracy

Often, a simple pixelwise accuracy reflecting the fraction of correct labels to total labels is used to assess the quality of segmentations. However, this can be misleading, since nanoparticles in electron microscope images generally comprise a small portion of an image, skewing a pixelwise accuracy in favour of the more numerous background pixels. This issue can be resolved using a confusion matrix, which displays true and false positive and negative rates, or a combination of these such as precision, recall or balanced accuracy. Precision is the fraction of classifier‐labelled particle pixels that are truly particle pixels, also known as positive predictive value (PPV). Recall is the fraction of total particle pixels that are correctly labelled as particle pixels by the classifier, also known as sensitivity. Precision and recall are therefore defined as

(3) $$Precision=\frac{tp}{tp+fp}\;Recall=\frac{tp}{tp+fn},$$

where *tp* is the number of true positives, *fp* is the number of false positives and *fn* is the number of false negatives. Precision and recall are often used in tandem as one of these measurements alone does not account for both false positives and false negatives when determining accuracy. Balanced accuracy is the weighted accuracy of the true‐positive rate (TPR) and true‐negative rate (TNR), that is, the average of the correctly labelled particle pixels and correctly labelled background pixels, expressed as

(4) $$\mathrm{Balanced}\;\mathrm{accuracy}=\frac{\left(\mathrm{TPR}+\mathrm{TNR}\right)}{2}.$$

To accurately assess filter kernel effectiveness and their similarity, the two‐sample Kolmogorov–Smirnov (2SKS) test^37^ and the Pearson's correlation coefficient^38^ (PCC) are used. The 2SKS test records the largest gap between the two cumulative distributions of filter kernel intensities of particle and background pixels. Hence, the higher the 2SKS statistic, the more effectively the filter kernel separates particle from background pixels. The 2SKS is defined as

(5) $$2SKS_{n,m}=\begin{array}{c} sup\\ i \end{array}\left|F_{1,n}\left(i\right)-F_{2,m}\left(i\right)\right|,$$

where ${}_{i}^{sup}$ is the supremum of *i*, and $F_{1,n}\left(i\right)$ and $F_{2,m}\left(i\right)$ are the distribution functions of particle and background pixels. The subscripts 1 and 2 refer to the two samples to be tested and *n* and *m* refer to the sizes of the first and second samples respectively.

The PCC measures the correlation of two filter kernels on a sample of pixels from the nanoparticle images. A PCC value of 1 indicates a perfect positive correlation while −1 indicates a perfect negative correlation, with 0 showing no correlation. The absolute PCC is used here, as a perfect negative correlation simply implies inverted labelling of the pixels, which does not contribute additional information to the classifier.

(6) $$PCC_{X,Y}=\frac{n\left(\Sigma \mathrm{xy}\right)-\left(\Sigma x\right)\left(\Sigma y\right)}{\sqrt{\left(n\Sigma x^{2}-{\left(\Sigma x\right)}^{2}\right)\left(n\Sigma y^{2}-{\left(\Sigma y\right)}^{2}\right)}},$$

where *x* and *y* are pixel samples from two filter kernels and *n* is the number of samples in both *x* and *y*.

The effectiveness and similarity tests, 2SKS and PCC, respectively, were both run on all data sets using their ground truths as the segmentation masks. The confusion matrices and accuracies were found with classifiers using the default parameters seen in Table 1. The specific parameters noted are: σ (the standard deviation used in the Gaussian and difference of Gaussian kernels), high_σ (the standard deviation of the second Gaussian used in the difference of Gaussian method) and disk_size (the size of the kernel used for median, minimum and maximum filter kernels).

**TABLE 1:** Parameters used for trainable segmentation on each data type

Image type	Image size (px)	Typical feature size (px)	Σ (px)	high_σ (px)	disk_size (px)
Pt ADF	1024	30	1	16	20
PdPtNiAu ADF	1024	50	1	16	20
PdC TEM	2048	50	4	64	20
AuGe TEM	2048	100	4	64	20

Global and local thresholding methods were also used to segment images in ParticleSpy, to compare against trainable segmentation. The parameters used for thresholding are shown in Table 2.

**TABLE 2:** Global and local thresholding algorithms and parameters used on each image type

Image type	Thresholding algorithm	Rolling ball filter	Gaussian kernel size	Local filter kernel size	Watershed	Watershed seed separation	Watershed erosion	Inverted
Pt ADF	Combined local/global Otsu	158	8	45	FALSE	–	–	FALSE
PdPtNiAu ADF	Li	301	1	–	TRUE	40	10	FALSE
PdC TEM	Combined local/global Otsu	600	7	50	FALSE	–	–	TRUE
AuGe TEM	Combined local/global Otsu	600	7	50	FALSE	–	–	TRUE

The classifier analysis was carried out by training the classifier on the desired number of random pixels 10 times in order to average out variations arising from the choice of a small sample of pixels. Each of these trained classifiers was tested on 10 images, and the classifiers’ accuracy was measured against the ground truth, before the final accuracies were calculated from the 10 images, each segmented 10 times. This process was repeated for each classifier of interest, with the filter kernel parameters specified in Table 1. The standard array of filter kernels (Gaussian, difference of Gaussians, median, maximum, minimum, Sobel and sum membrane projection) were used for all tests of trainable segmentation accuracy.

Training and classification times reported here were measured on a Ryzen 5600X CPU with 16 GB of RAM at 3000 MHz. We note that training and classification times vary between different hardware configurations but are presented as a comparison between the classifiers used here.

### 2.4 Image sets

**FIGURE 3:**
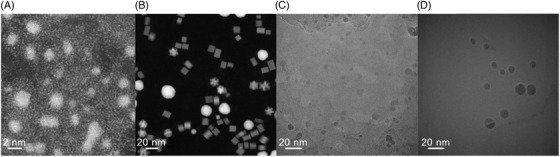
Example images from the 4 data sets used to test the trainable segmentation routines. (A) Pt ADF, (B) PdPtNiAu ADF, (C) PdC TEM and (D) AuGe TEM.

In order to test the accuracy and applicability of trainable segmentation, we used four sets of images (each set containing 10–30 images) that display different contrast, features and noise levels. Two sets of HAADF STEM images and two sets of TEM images were used to test the effect of differing contrast. The HAADF STEM sets included one of Pt nanoparticles at atomic resolution (referred to as Pt ADF) and another including a mixture of Pd, PtNi and Au nanoparticles (referred to as PdPtNiAu ADF, as used in Ref. 6). The TEM images are of Pd nanoparticles on a carbon support material and Au nanoparticles on a Ge support material; these are referred to as PdC TEM and AuGe TEM, respectively. Example images from these data sets are shown in Figure 3.

## 3 Results and discussion

### 3.1 Pixel distribution analysis and filter kernel selection

To compare the effectiveness of each filter kernel, 2SKS tests were used to evaluate the effectiveness of each filter kernel, while PCC tests were used to analyse the similarity of the filter kernels on each type of image.

#### 3.1.1 Filter kernel effectiveness

**FIGURE 4:**
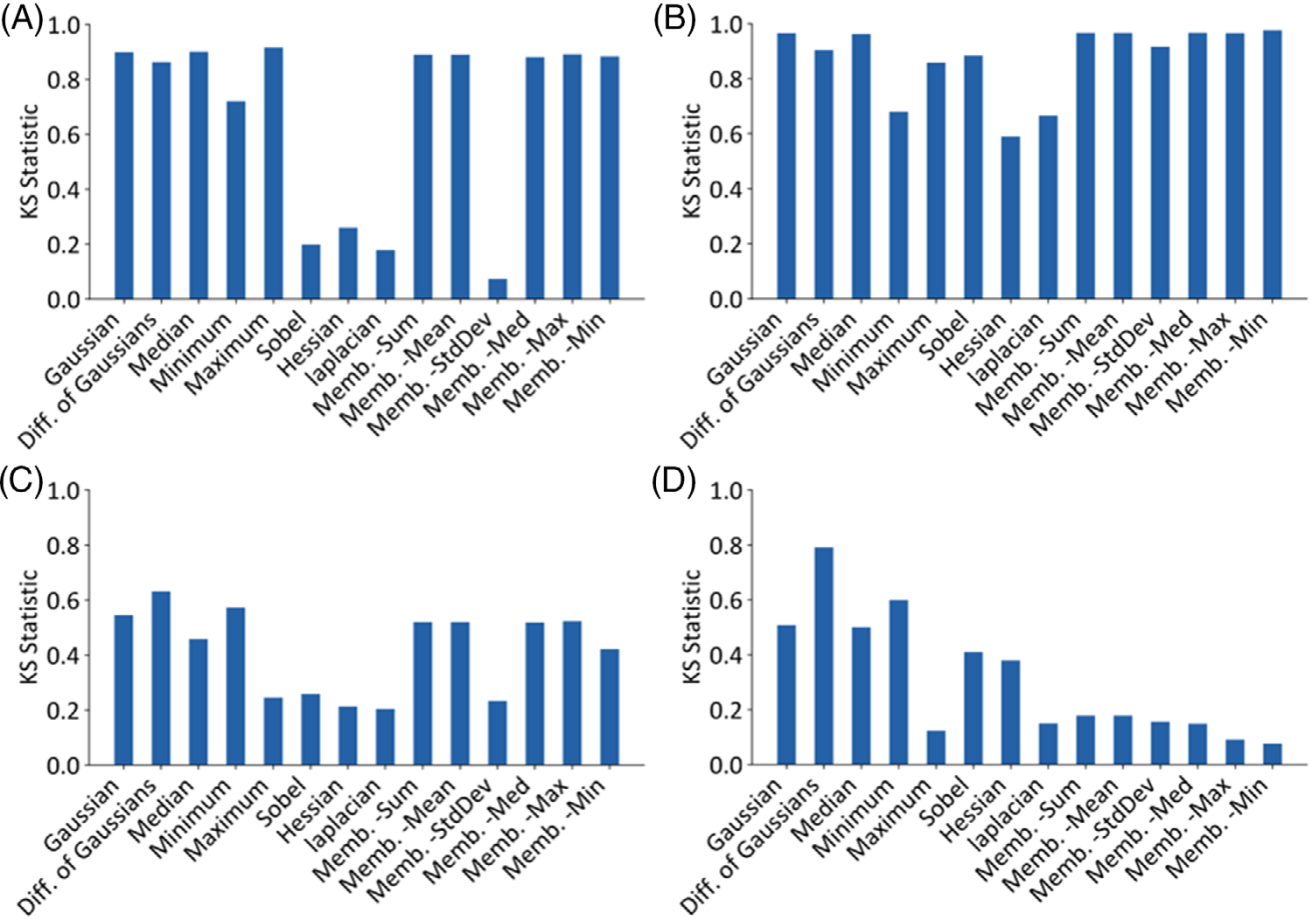
2–Sample Kolmogorov–Smirnov statistics between particle and background pixel distributions for each data set. (A) Pt ADF, (B) PdPtNiAu ADF, (C) PdC TEM and (D) AuGe TEM images. High 2SKS Statistic values indicate larger separations between the distributions.

Figure 4 shows the 2SKS statistic for each of the image types. As expected, the 2SKS statistic of the PdPtNiAu ADF data set shows many filter kernels are effective at distinguishing the pixel sets due to the high contrast and clear boundaries between the two sets of pixels (Figure 4B). Many filter kernels retain a 2SKS statistic above 0.8 for the Pt ADF data (Figure 4A), suggesting that pixel sets can be clearly distinguished using a majority of filter kernels. However, for the two TEM data sets, only the difference of Gaussians has a 2SKS statistic above 0.6 (Figure 4C and D). The lower contrast between particles and background in the TEM images decreases the effectiveness of all filter kernels. However, certain filter kernels do possess a higher 2SKS value for both TEM image sets and therefore careful choice of which filter kernels to use is important.

The best performing filter kernels for all images included many of the intensity‐based filter kernels, particularly the Gaussian, difference of Gaussians and median kernels. It is also interesting to note that the maximum filter kernel performs better on the ADF images than the minimum, which performs better on the TEM images. This is due to the particles’ intensities, which compose a small percentage of the image, being brighter or darker than the background of the image respectively, which allows the minimum or maximum filter kernels to isolate particle pixels more effectively.

In all cases the Laplacian and Hessian filter kernels perform poorly, not discriminating between particle and background pixels, most likely due to the uniform texture in most data sets. The exception to this is the AuGe TEM images, where the Hessian filter kernel performs comparatively well against the other filter kernels (it has the 6th highest 2SKS score, vs. the 11th, 13th and 14th highest scores on the other data sets). The AuGe TEM images qualitatively contain a greater degree of lattice fringes in each particle that may contribute to the effectiveness of the Hessian in this case. It is also possible that the very flat background in the AuGe TEM images means that there are smaller variations due to background contrast, which aids in discrimination of the particles. While the use of a Hessian filter may be useful where texture is a clear discriminating feature of particles, that is not the case here and therefore we do not include Hessian or Laplacian filters beyond this section.

All membrane projection filters perform similarly, barring the standard deviation filter which typically performs poorly compared to the other membrane projections (lowest in each data set other than the AuGe). The Sobel filter also performed poorly on the Pt ADF image (less than 0.2 2SKS score), due to the ill‐defined boundaries between the particle and background.

The 2SKS statistic could be used to automatically pick the most effective filter kernels and filter kernel parameters on a specific set of images, with 2SKS values above a given value (0.5 is suggested) selectively used to classify the image with the user‐labelled pixels. Automatic filter kernel selection using 2SKS would greatly streamline the classification process and will be developed in ParticleSpy in the future.

#### 3.1.2 Filter kernel similarity

**FIGURE 5:**
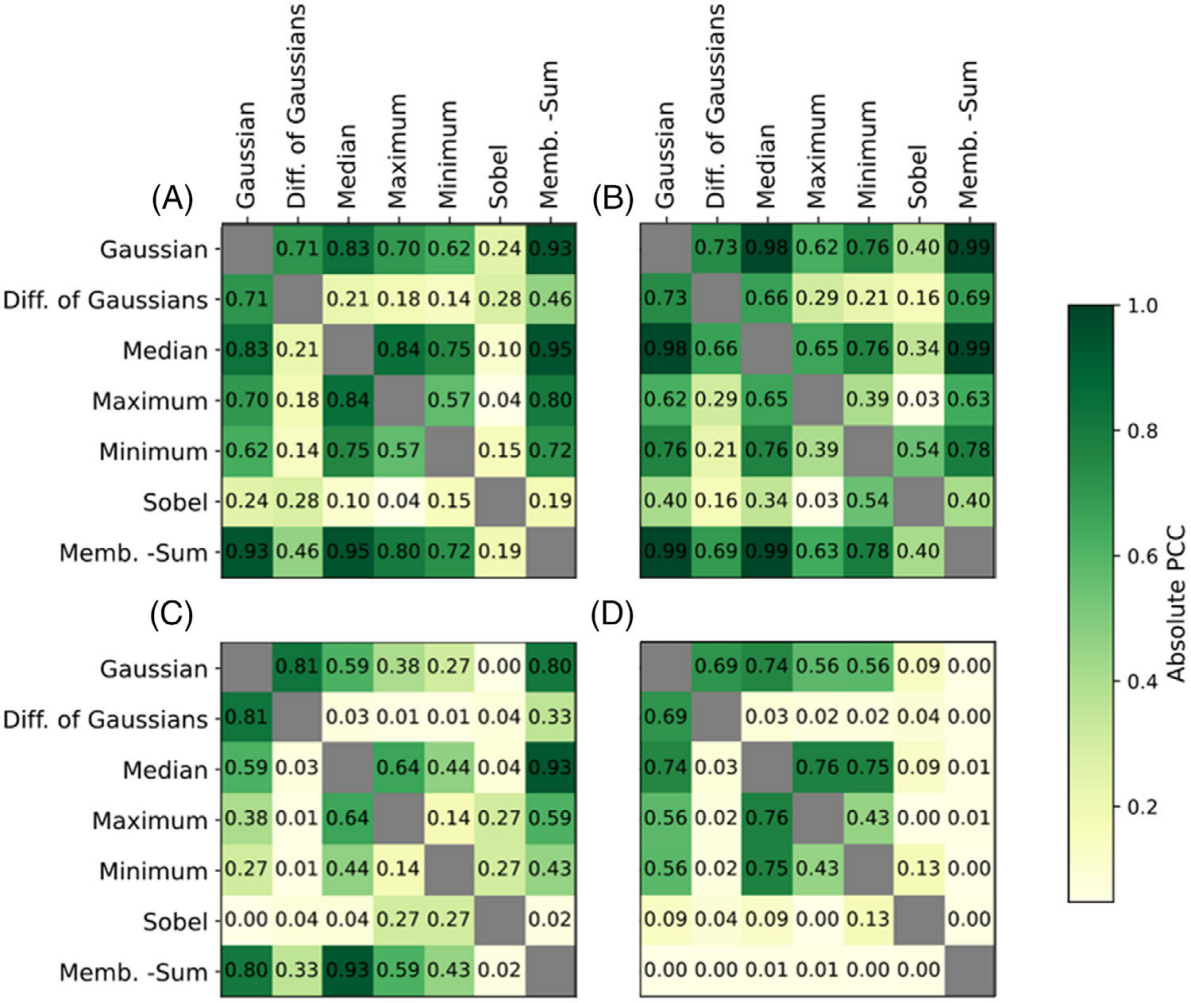
Pearson's correlation coefficient matrices between filter kernel pairs for (A) Pt ADF, (B) PdPtNiAu ADF, (C) PdC TEM and (D) AuGe TEM images. High PCC values indicate filter kernels sharing similar distributions.

The similarity of kernels measured using PCC is shown in Figure 5 and Figure S1, for the standard array of kernels and for the membrane projections respectively. Each value in both plots is the PCC of the two corresponding filter kernels on each axis.

All intensity‐based kernels (Gaussian to minimum) are relatively similar across all data sets (as shown in Figure 5), with all values above 0.5, except in the PdC TEM data set. The Sobel filter kernel is the only edge‐detecting kernel in the standard array, giving it a very low PCC with all other filter kernels (0.4 or lower) and making it an important kernel if it possesses a high 2SKS statistic. Overall, the TEM images display lower PCC values than the ADF images. This is due to the same factors that cause the lower effectiveness of filter kernels on the TEM images, namely that contrast is lower in the TEM images and therefore it is more difficult to distinguish between particle and background. This is not to suggest that two highly effective filter kernels must also be highly correlated. For example, the difference of Gaussians and minimum filter kernels are both highly effective on the TEM images sets while sharing a low PCC.

For all data sets (apart from AuGe TEM), the membrane projections show very high PCCs (values above 0.9), excluding the standard deviation (Supporting Figure S1). On the ADF data sets, the inclusion of more than one membrane projection feature is unnecessary due to their similarity. The feature set may prove more useful than just the mean feature on some TEM data sets, where PCC values are lower than 0.8.

#### 3.1.3 Default filter kernel selection

The filter kernel array of Gaussian, difference of Gaussians, median, maximum, minimum, Sobel and sum membrane projection was chosen as a standard array of kernels, which provided high 2SKS values and low PCC values for all tested data sets, and only includes one of the six typically degenerate membrane projections. The use of more filter kernels does not affect the time taken by the classifier, as classifiers need only set boundaries in the arbitrary dimensions of the feature set. However, fewer filter kernels take less time to generate features for each image, which saves time in the overall classification process. The parameters used by these filter kernels on each data set are given in Table 1 and were chosen to optimise the 2SKS values (as shown in Figure 4). Additional filter kernels could have been tested; however, the tested kernels were equivalent to or greater than the default selection in other trainable segmentation software packages.^23^, ^26^

### 3.2 Classifier analysis

The choice of an appropriate classifier can significantly improve the segmentation of a data set. Beyond pixelwise accuracy, it is important to consider whether high positive predictive values are preferred, or high recall values are more desirable in segmentation. This is essentially a choice between under‐segmentation and over‐segmentation. It is also important to assess the number of pixels required by a classifier for accurate segmentation, to ensure that a sufficient number of pixels are labelled by the user to produce accurate results. The following sections will examine the choice of classifier and its effect on segmentation accuracy, the minimum number of pixels required for classifiers to segment effectively and the time required to train each classifier.

#### 3.2.1 Classifier precision and recall

**FIGURE 6:**
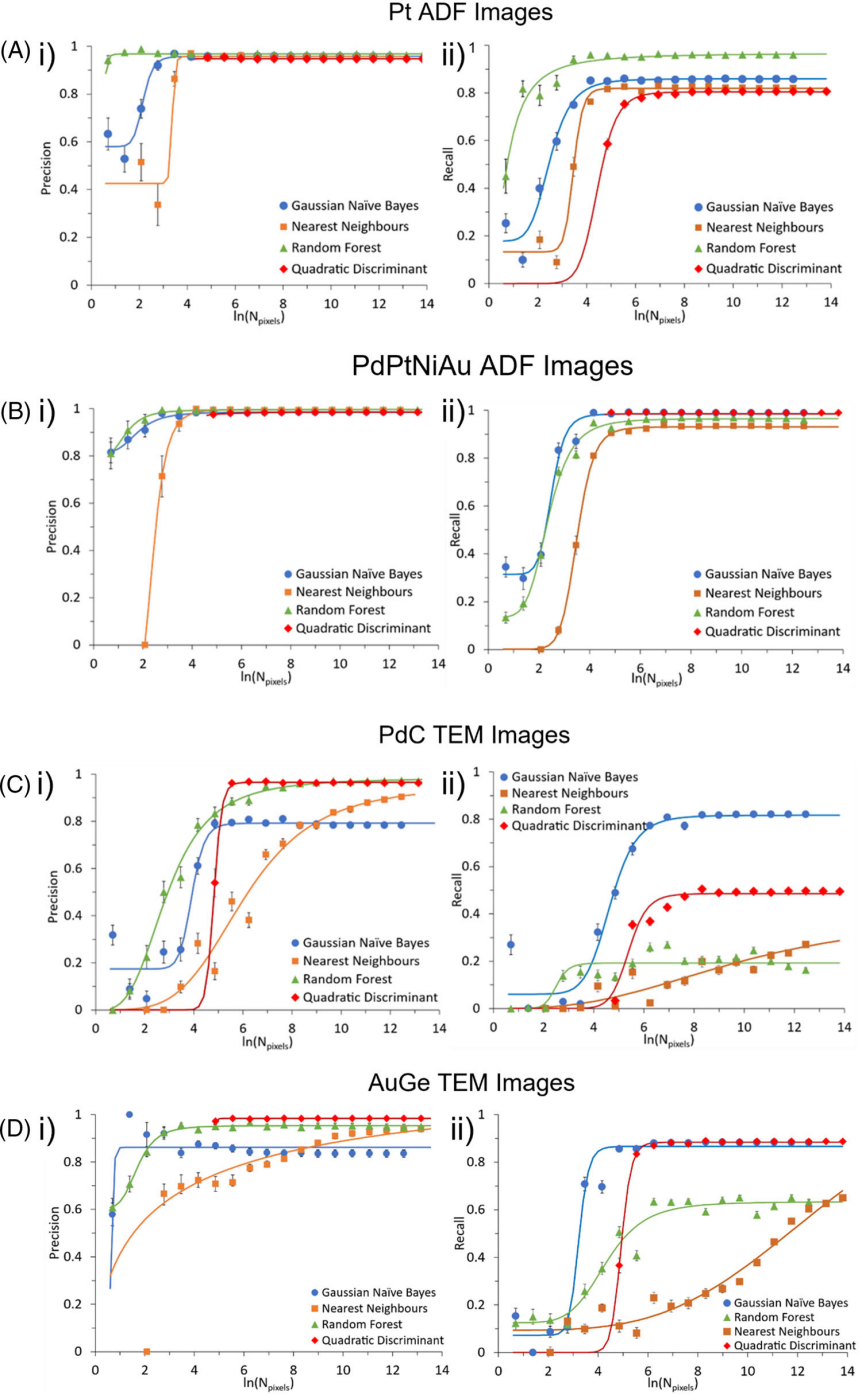
(i) Precision and (ii) recall for the (A) Pt ADF, (B) PdPtNiAu ADF, (C) PdC TEM and (D) AuGe TEM data sets plotted against the natural logarithm of the number of pixels used to train the classifier. This was done for four selected classifiers. Error bars are the standard error and are shown where significant. Fitted sigmoid curves are for visual aid only.

We have tested the four classifiers described in Section 2 on each of the four sets of images (results presented in Figure 6) in terms of their precision and recall. The plots of Figure 6 display precision and recall values as a function of the number of pixels used in training.

We first note that in all data sets, the QDA classifier provided a high precision metric (above 0.9). However, the recall metric for QDA is not always high (0.8 for Pt ADF and 0.5 for PdC TEM). This suggests that QDA has a tendency to under‐segment the images; that is it assigns particle pixels as background pixels. In the two cases of PdPtNiAu ADF and AuGe TEM, the QDA retains a high precision and recall (above 0.9). In these two image sets, there is little variation in the background, whereas the other two image sets have strong background variations that appear to contribute to under‐segmentation. We therefore suggest that the QDA classifier is a good choice for any data set in which a smooth background is present.

The Guassian Naïve Bayes classifier typically provides the highest recall values, but it returns the lowest precision values for both sets of TEM images. This suggests that in the case of low contrast (as in TEM images), the Gaussian Naïve Bayes classifier tends to under‐segment particles. Nevertheless, the Gaussian Naïve Bayes classifier has relatively good performance for all data sets and therefore could be used as a catch‐all classifier when unsure of which to use.

As with the QDA classifier, high precision values are obtained when using the Random Forest classifier (above 0.9) for all data sets. However, when applied to the TEM images, low recall values are found (0.6 and 0.2). This suggests that the Random Forest classifier performs well with high contrast images, but under‐segments particles when contrast is low. Therefore, the Random Forest classifier is recommended for use with ADF images but not TEM images.

The nearest neighbours classifier typically has the lowest recall value for all data sets. However, as will be described in the next section, values for the TEM images are still increasing with number of pixels at the maximum number of pixels used in this study (approximately 1,000,000 pixels). Using this classifier therefore tends to result in under‐segmentation, particularly for TEM images, but may improve if trained on many images.

#### 3.2.2 Minimum pixel requirements

It is also important to consider the minimum number of pixels required for a given data set to produce an accurate segmentation, as the time spent manually labelling pixels for training can be reduced accordingly. The number of pixels required will vary considerably for different classifiers, and so it is important to understand the number of pixels that each classifier requires for segmentation. In this section, analysis of the minimum pixels needed for each data set is presented, where the number of pixels is the total of particle and background pixels from a random selection of the labelled image pixels.

Given how the nearest neighbours and QDA classifiers operate, very low numbers of labelled pixels cannot be used for these classifiers. The nearest neighbours classifier requires more pixels than its parameter *n_samples*, while QDA requires a minimum number of both particle and background pixels to train, meaning it could not consistently train on small numbers of pixels without errors (we have used 75 pixels of each as a starting point).

All classifiers on the Pt ADF data (Figure 6A) reach their maximum accuracy by 2000 pixels (where ln(2000) = 7.60), with the random forest classifier reaching its maximum accuracy at around 1000 pixels (where ln(1000) = 6.90). For all the data sets, the precision reached its maximum in fewer pixels than the recall. This effect is due to the classifier initially under‐segmenting the image, which gives a high precision, but low recall values. As the classifier trains using more pixels, the boundaries between the particle and background pixels are refined, and the number of false‐negative drops, giving a higher overall accuracy.

The PdPtNiAu ADF data set was the fastest to achieve accurate classification (Figure 6B), requiring around 60 pixels (ln(60) = 4.1) to reach near maximum precision and recall for the Naïve Bayes classifier, with the remaining classifiers reaching their maximum balanced accuracy by 4000 pixels. All classifiers had a high precision (greater than 0.95) at larger numbers of pixels.

The PdC TEM data set is one of the hardest to train, and as a result produces the greatest variability in the performance of the classifiers (Figure 6C). The Random Forest classifier reaches its maximum accuracy at 16,000 pixels (ln(16,000) = 9.7), while the nearest neighbour classifier does not reach its maximum accuracy by 1 megapixel. The other two classifiers perform better; the Naïve Bayes and the QDA classifier both reach their maximum accuracies by 4000 pixels and achieve higher recall values overall. The choice between these two classifiers for this data set would be determined by the requirements of the segmentation masks, rather than simply the balanced accuracy.

The AuGe TEM data set (Figure 6D) shows high precision for all classifiers. The random forest and Naïve Bayes classifiers reach their maximum accuracies by 8000 and 1000 pixels, respectively. The nearest neighbour classifier does not reach its maximum before 1 megapixel. In this data set, the most accurate classifier is the QDA classifier, achieving its maximum accuracy at around 1000 pixels. While it cannot classify images for the small number of pixels at which the Naïve Bayes classifier is accurate, QDA is more precise than Naïve Bayes, with fewer false negatives. The nearest neighbours classifier appears to trend upward at the limit of the maximum number of pixels used for training. However, it does not surpass, or equal the accuracy of the QDA classifier even at a larger number of pixels.

#### 3.2.3 Classifier accuracy

**FIGURE 7:**
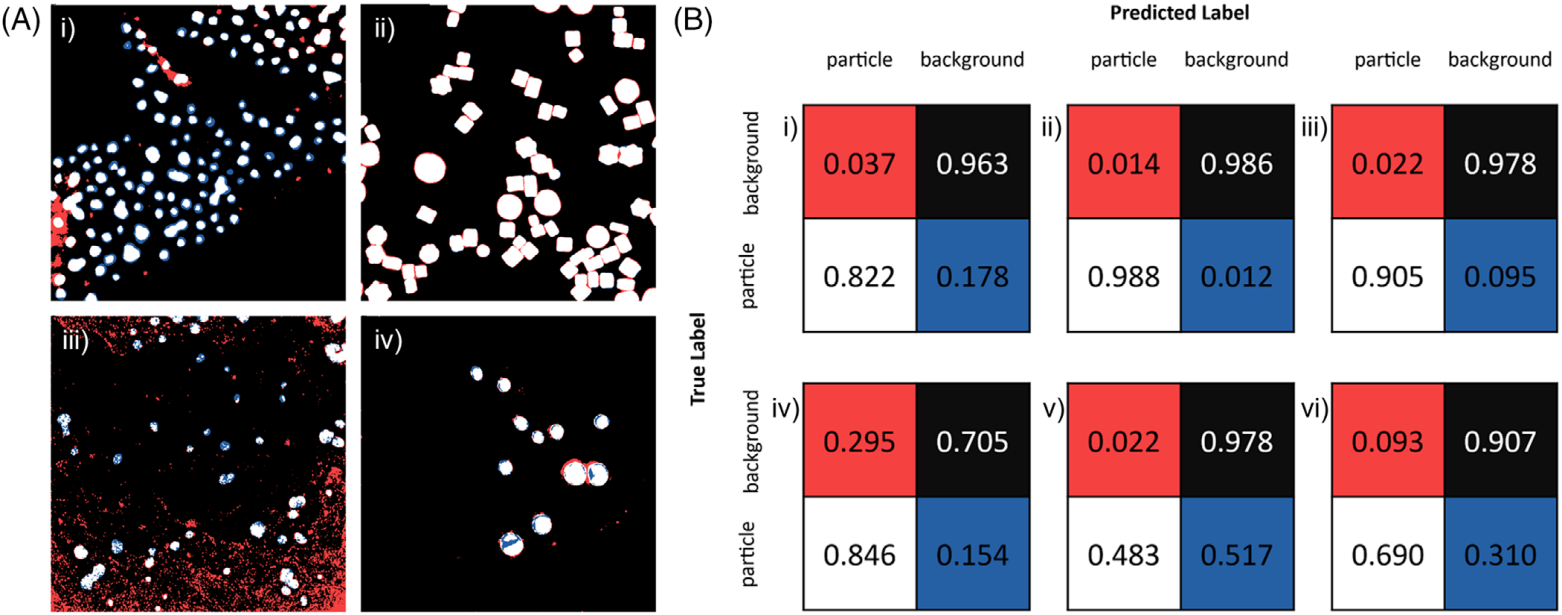
(A) Overlaid trained segmentation masks on ground truth segmentations for (i) Pt ADF using the Random Forest Classifier, (ii) PdPtNiAu ADF using the Naïve Bayes Classifier, (iii) PdC TEM using the Naïve Bayes Classifier and (iv) AuGe TEM using the QDA Classifier. Black and white regions indicate true negative and true positive regions, respectively. Red and blue regions indicate false positives and false negatives, respectively. (B) Confusion matrices for the data sets: (i) Pt ADF using the random forest classifier, (ii) PdPtNiAu ADF using the Naïve Bayes classifier, (iii) AuGe TEM using QDA, (iv) PdC TEM using the Naïve Bayes Classifier, (v) PdC TEM using the QDA classifier and (vi) PdC TEM trained on five images using the Naïve Bayes classifier. The red, black, white and blue squares represent false‐positive, true‐negative, true‐positive and false‐negative values, respectively.

The most effective classifiers (in terms of their balanced accuracy) for each data set are presented in Figure 7 as a classified mask overlaid on a ground truth image, together with the confusion matrices for each data set (and two additional matrices for the PdC TEM data set). In this section, we describe the use of the most accurate classifier on each data set.

The Pt ADF data set was typically accurately segmented by most classifiers. The best of these was the random forest classifier, which recorded a balanced accuracy of 89.2%, trained the classifier in 19 s per megapixel, and classified subsequent images at a rate of 13 s per megapixel. Figure 7A(i) shows that some parts of the image have been over segmented, showing false positives in high intensity areas, such as on the left‐hand side. Conversely, many of the particles, particularly at the centre of the image, have been under‐segmented (as revealed by false‐negative pixels). In this particular case, where image intensity varies within each particle, it is therefore critical to take care in training classifiers to include pixels describing all image intensities of both particle and background.

The PdPtNiAu ADF data set is best segmented using the Gaussian Naïve Bayes classifier, which gave a balanced accuracy of 98.7%. The classifier took 14 s per megapixel to train, and 13 s per megapixel to classify the remaining images. This high accuracy is reflected in Figure 7A(ii), which shows almost no under‐segmentation of the particles, with a small amount of over‐segmentation around most particles. The excellent performance is due to the high contrast of the images, which makes all segmentation methods easier to implement accurately.

The least accurate and most inconsistent set of trainable segmentation results was found from the PdC TEM data set. This is due to the very low contrast between background and particle pixels, making even manual segmentation difficult. The Naïve Bayes classifier gives the highest balanced accuracy (Figures 7A(iii) and 8B(iv)), with a balanced accuracy of 77%, training the classifier in 14 s per megapixel and classifying each subsequent image at 12 s per megapixel. However, there are many false positives produced by this classifier, compared to QDA (Figure 7B(v)), although the positive predictive value from Naïve Bayes was almost twice as high as that of QDA (0.846 vs. 0.483). Many particles are almost fully segmented by the Naïve Bayes classifier, but have regions of false negatives at the centre of the nanoparticles due to higher pixel intensities falling outside of the particle classification boundary.

**FIGURE 8:**
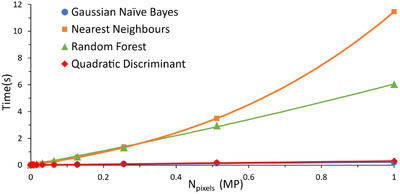
Time to train classifiers plotted against number of pixels, in megapixels, used to train the classifier. Fitted curves are linear for Naive Bayes and QDA, second‐order polynomial for nearest neighbours, and third‐order polynomial for the Random Forest classifier.

The PdC TEM data set benefits from multi‐image training, where training on five ground truth images (Figure 7B(vi)) reduces the number of false positives but increases the number of false negatives, producing an overall balanced accuracy of 79%. The benefits of this multi‐image training do not arise from the increased number of samples, but from the wider variety of pixel samples that many images provide. This may be due to an underrepresentation of different types of particles in the initial single image used for training, which is solved by including a larger number of images.

The best performing classifier on the AuGe TEM data set was the QDA classifier, with a balanced accuracy of 94%. This classifier took 14 s per megapixel to train and the same time to classify each image. Figure 7D shows the segmentation of one image overlaid on the ground truth. It is clear that some regions around particles have been over‐segmented and, notably, many particles are also under‐segmented at their centre. This is reflected in the confusion matrices, which show that around 10% of particle pixels are mislabelled. These results were produced from training on only one image; however, additional training did not improve the segmentation accuracy, in contrast to the PdC TEM data set. We suggest this is because the AuGe TEM images are less complicated than the PdC TEM images, with fewer variations in the support across different images. This is an important point to note; if there are significant variations between images in a set, training on multiple images will be necessary to capture these different features.

The Gaussian Naïve Bayes and the QDA classifier perform identically on the PdPtNiAu ADF data set. As discussed in Section 2, this should be true if the two distributions defined are well separated. Therefore, this result is unsurprising for this case in which particle and background pixels can be easily distinguished simply by their intensity.

#### 3.2.4 Classification time

There is little existing analysis of classifiers used in trainable segmentation, and as these comprise a large part of the trainable segmentation process, they are important to consider. Previous users of ImageJ's trainable segmentation have not detailed their choice of classifier, arguably the most important factor in segmentation, but have noted slow classification times that may result due to classifier choice.^29^ A more detailed analysis of different classifiers and their performance is therefore generally useful and will be presented here.

The time to classify was equal over all data sets, so only one data set is presented (Figure 8). Both the Naïve Bayes and QDA classifier have very low training times, and a linear relationship between the training time and the number of pixels used for training. This indicates a big‐O notation of O(N) where *N* is the number of pixels used for training, as the Naïve Bayes classifier and QDA classifier only need to calculate the probability for each pixel's set of features. The nearest neighbours classifier takes the longest to train for large numbers of pixels, with a big‐O notation of $O(N^{2})$, as each data pixel must have its nearest neighbours calculated and stored after checking through every other pixel. The random forest classifier lies between the training time of nearest neighbours, and QDA and Naïve Bayes at this range of pixels. It has a big‐O notation of $O(N^{3})$, meaning with a sufficiently large number of pixels its training time would surpass nearest neighbours; however, this would only occur at a considerably larger number of pixels than that needed for accurate training. Note that these Big‐O notations are empirical, rather than extrapolated from the classifier's code. These results support our suggestion to use a Gaussian Naïve Bayes classifier if unsure of which to use, as its training time will be comparatively short in most cases.

### 3.3 Comparison to global and local thresholding

Many contemporary studies still use global or local thresholding methods to segment images,^6^ or as part of a hybrid thresholding algorithm.^13^ A more versatile and accurate method such as trainable segmentation would be useful in these cases.

**FIGURE 9:**
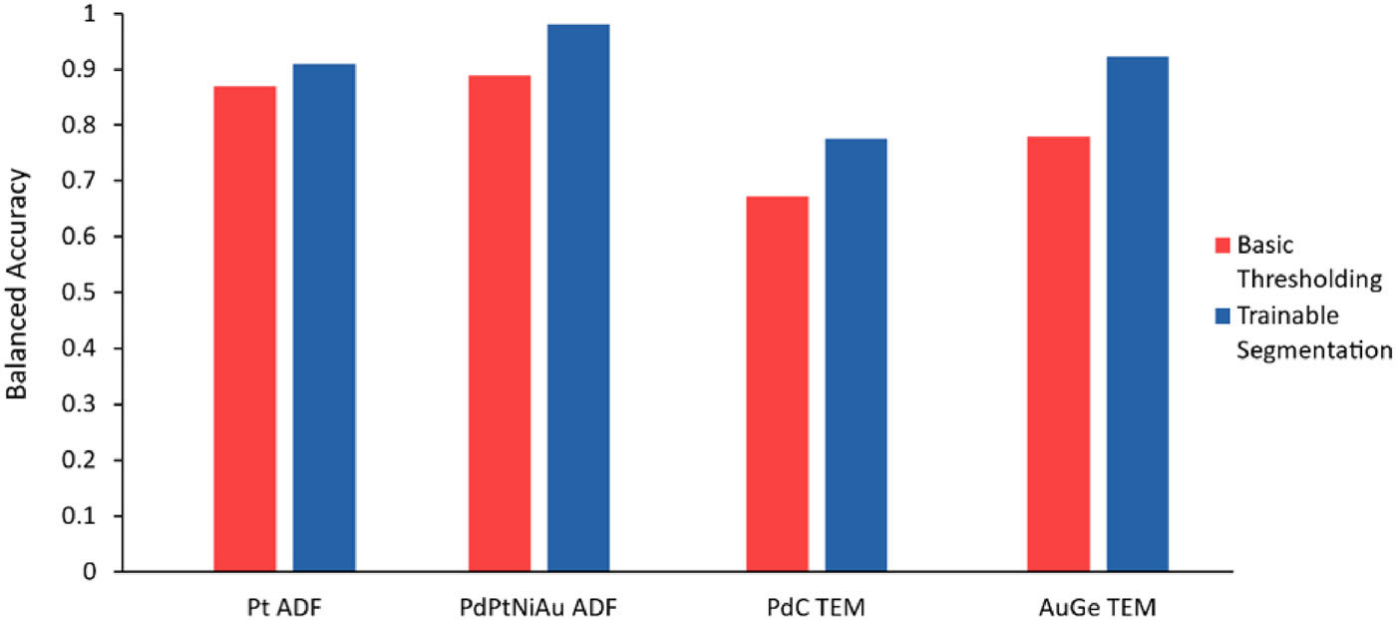
Balanced accuracy of basic thresholding and trainable segmentation on all four image types using 10 sample images.

A comparison of the balanced accuracy of global or local thresholding and trainable segmentation is shown in Figure 9. Global or local thresholding algorithms were selected to maximise the balanced accuracy for each data set by testing each method available in ParticleSpy; that is the optimally performing thresholding algorithms and parameters were chosen for each image set (as displayed in Table 2). These thresholding algorithms were compared to the optimally performing trainable segmentation for each data set. In all cases, the trainable segmentation accuracy is higher than the best‐performing global or local thresholding available via ParticleSpy. It is also important to note that basic thresholding still requires user input to refine the parameters of the threshold and to select an appropriate thresholding algorithm.

## 4 CONCLUSIONS

Trainable segmentation offers accurate segmentation across a variety of image types including high‐resolution TEM images, and its implementation in ParticleSpy includes a wide variety of filter kernels and classifiers. We have analysed each of these filter kernels and classifiers to demonstrate their use cases, as well as their advantages and drawbacks. Our analyses included the effectiveness and similarity of all ParticleSpy filter kernels, and the number of pixels required for accurate training of each classifier. These parameters were used to determine the default selection of filter kernels in ParticleSpy as a baseline for users of trainable segmentation. Specifically, we use Gaussian, difference of Gaussians, median, maximum, minimum, Sobel and sum membrane projection filters kernels as the default selection in ParticleSpy. Additional filter kernels can be added in specific cases, such as when image texture is a key determining feature.

We have demonstrated the most effective classifier for each data set, along with their training speeds per megapixel. Specifically, we have found that a Random Forest classifier performs best for high‐contrast ADF images, but that QDA and Gaussian Naïve Bayes classifiers perform better for low‐contrast TEM images. A Gaussian Naïve Bayes classifier was found to be the best choice in terms of all‐round performance (comparatively high balanced accuracy and a short training time) and is the best equipped to deal with large variations in background intensity.

The techniques described allow large data sets of nanoparticle images to be processed and analysed with minimal user input, automatically selecting appropriate default filter kernels. For this reason, trainable segmentation, as implemented in ParticleSpy, has the potential to significantly enhance segmentation of electron microscope images of inorganic nanoparticles, providing a more accurate measurement of size and shape parameters that play an important role in the physical and chemical particle properties of these particles. ParticleSpy is a freely available open‐source python package for particle segmentation,^39^ the source code, documentation and example notebooks can be found at https://github.com/ePSIC‐DLS/particlespy.

## Supporting information

Supporting Figure S1. Pearson's correlation coefficient matrices between membrane projection filter kernel pairs for (A) Pt ADF, (B) PdPtNiAu ADF, (C) PdC TEM and (D) AuGe TEM images. High PCC values indicate filter kernels sharing similar distributions.
