# Supplementary material for: Trainable segmentation for transmission electron microscope images of inorganic nanoparticles
Source: J Microsc. 2022 May 11;288(3):169–84. doi: 10.1111/jmi.13110 (PMC10084002; doi:10.1111/jmi.13110)
Supplement: Supplementary file 1 — Supporting Figure S1. Pearson's correlation coefficient matrices between membrane projection filter kernel pairs for (A) Pt ADF, (B) PdPtNiAu ADF, (C) PdC TEM and (D) AuGe TEM images. High PCC values indicate filter kernels sharing similar distributions. [file JMI-288-169-s001.docx]

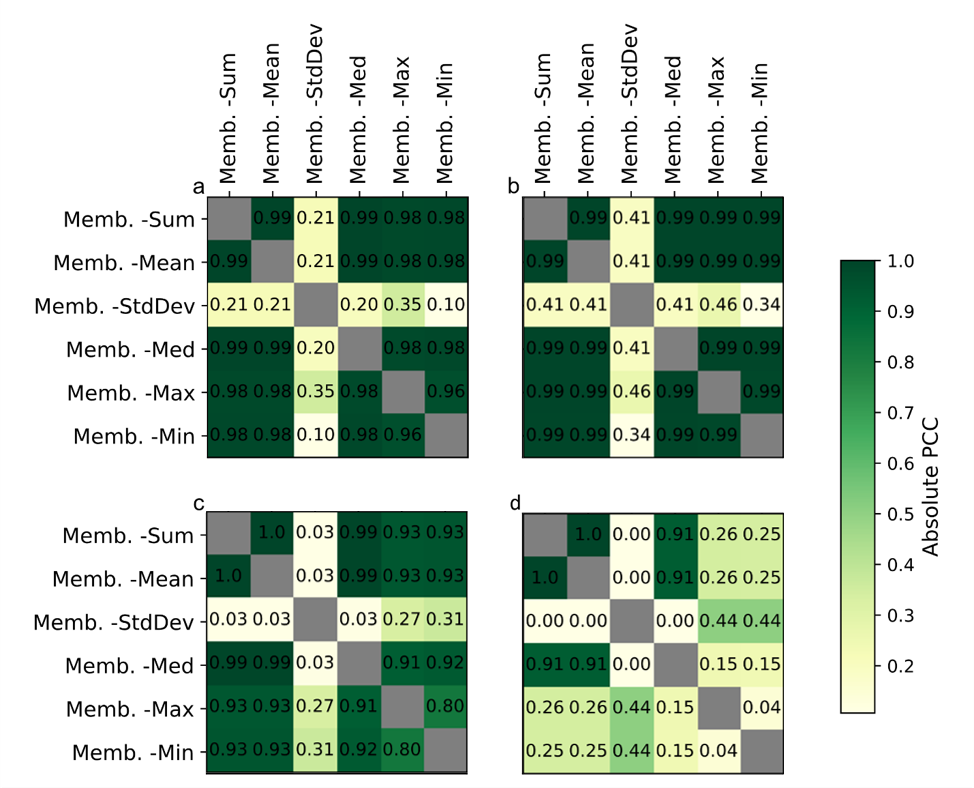


Supporting Figure 1. Pearson Correlation Coefficient matrices between membrane projection filter kernel pairs for (a) Pt ADF, (b) PdPtNiAu ADF, (c) PdC TEM and (d) AuGe TEM images. High PCC values indicate filter kernels sharing similar distributions.
